# The Application of Functional Magnetic Resonance Imaging in an Infant Rat Model of Irritable Bowel Syndrome

**DOI:** 10.1155/2014/473846

**Published:** 2014-07-01

**Authors:** Xueping Zhu, Xiaoli Zhu, Weichang Chen, Jianhua Chen

**Affiliations:** ^1^Department of Neonatology, Soochow University Affiliated Children's Hospital, Suzhou 215003, China; ^2^Department of Intervention, The First Affiliated Hospital of Soochow University, Suzhou 215006, China; ^3^Department of Gastroenterology, The First Affiliated Hospital of Soochow University, Suzhou 215006, China; ^4^Department of Medical Imaging, The First Affiliated Hospital of Soochow University, Suzhou 215006, China

## Abstract

The aim of this study was to use functional magnetic resonance imaging (BOLD-fMRI) to investigate the activated region associated with visceral pain in the brains of infant rats in a model of IBS. Sixteen newborn rats were randomized into an IBS model group and a control group. Those in the IBS group were separated from their mothers and were mechanically immobilized and had rectal sensitization with mustard essential oil for 1 week. The control group had no treatment. After 2 weeks, balloon catheters were inflated with 5 or 10 mL of air in the rectums of both groups. BOLD-fMRI was performed and the data analyzed by imaging software. In the IBS model group, rectal stimulation with 5 mL air distension activated the anterior cingulate cortex, insula cortex (IC), prefrontal cortex (PFC), and thalamus, while 10 mL air significantly activated the ACC, IC, PFC, and thalamus in the model, but not controls. IBS model group was hypersensitive to visceral stimulation by rectal balloon inflation. The major brain areas participating in visceral sensation included the IC, PFC, and thalamus.

## 1. Introduction

Irritable bowel syndrome (IBS) is one of the most commonly seen functional diseases of the gastrointestinal tract, clinically manifesting as intermittent or continuous attacks of abdominal pain, abdominal distension, and abnormalities of bowel habits and stool [1–4]. There is currently no biochemical, histopathological, or radiological diagnostic test for IBS. The diagnosis of IBS is currently based mainly on symptom assessment [5]. The pathophysiology of this disease remains poorly understood. Hypersensitivity of visceral sensation has been postulated to be one of the pathophysiological mechanisms in the development of IBS [6, 7]. This is possibly related to a functional abnormality of the brain-gut nerve axis. However, the specific contribution of the brain-gut nerve axis components to hypersensitive responses in IBS remains unclear [8]. Many studies have demonstrated that many areas and nuclei in the brain are involved in the regulation of movement in the gastrointestinal tract [9–11]. Brain structures most likely to be involved are those surrounding the ventricles, such as the fornix, amygdala, cingulate, and hypothalamus. The central nervous system plays an important role in the regulation of IBS [8].

The aim of the current study was to use blood-oxygen-level dependence functional magnetic resonance imaging (BOLD-fMRI) to investigate the level and the distribution of the central activation areas of visceral pain in the brains of infant rats in a model of irritable bowel syndrome.

## 2. Materials and Methods

### 2.1. Model Animals and Randomization

Sixteen newborn SD rats at two days of age, regardless of sex, weighing from 5 to 6 g, were purchased from the Animal Center in Soochow University. They were randomized into two groups using a simple number table. Ten rats in the IBS model group [12] underwent separation from their mothers about 3 hours daily from postnatal days 2 to 21 and were subjected to mechanical immobilization and daily rectal stimulation with mustard essential oil between 8 and 15 days [11]. After the intervention, rats were allowed to rest for 2 weeks. Six newborn rats in the control group were separated from their mothers after being fed by their mothers for 20 d. No other intervention was performed in the control group. The environment parameters included temperature at 23 ± 1°C, humidity at 45–55%, free access to laboratory chow and drinking water, and a circadian cycle of a 12 h day and 12 h night. The study protocol was approved by the Animal Care Committee of the Children's Hospital Affiliated with Soochow University.

### 2.2. Colorectal Distension and BOLD-fMRI in Infant Rats

On the 30th day, rats fasted for 8 to 12 h to reduce stool formation, but water was not restricted. The residual stool was evacuated as much as possible with slight stimulation of the anus before the experiment.

Infant rats were anesthetized with 40 mg/kg pentobarbital intraperitoneally. The infant rats were placed in a supine position and fixed on a plastic box containing processed pork to induce rats to enter head first into the MRI coils vertically. After anal lubrication with paraffin oil, a self-made distension balloon was inserted into the rectum to a distance of 4.0 cm from the anal verge. The balloon end was situated 1.0 cm from the anus and was fixed to the base of the rat tails with tape.

The distension balloon consisted of a 3F vasodilation balloon catheter with T branch (CORDIS Co.) connected to a 6 Fr Foley catheter (Suzhou Weikang Medical Apparatus Co., Ltd.). The distal end of the Foley catheter was fixed and sealed with the fingertips of latex gloves resulting in a length 4.0 cm. A 20 mL syringe was connected to the T branch.

A 1.5T Marconi superconducting MRI and standard quadrature wrist coil were used with a gradient field of 23 Mt/M, and the shear rate was 120 mT/m*·*ms. MR images were taken in sagittal slices with parameters of TR/TE = 400 ms/12 ms, matrix at 256 × 256, NEX = 2, FOV 10 cm, resolution at 3 mm, intervals at 0 mm, and 789 with a 90° flip angle.

Central activation was produced by stimulation with balloon distension in the rectum in sessions divided into 10 50-second intervals to record the number and frequency of stimulations. During stimulation, the balloon was repetitively inflated and deflated during which the volume of air was alternately 5 mL and 10 mL. In between stimulations, there was no inflation or deflation of the balloon.

### 2.3. Analysis of MR Statistics

The Marconi MR workstation processed the brain function signals using MEDx. The brain function of infant rats was first calibrated for the head position with the anatomical profile. Then, the signal intensity was unified for statistical tests. The between-group tests were conducted with an independent-sample *t*-test, and *P* < 0.05 was considered to be statistically significant. Finally, the functional images were merged with the anatomical profiles.

## 3. Results

The results from fMRI indicated that the neurons in the visceral pain-related brain areas were activated and appeared as more highly excited areas in IBS group than those of the control group (*P* < 0.0001). With the balloons inflated with 5 mL air in the rectum, the highly excited areas were located in the prefrontal cortex (PFC 6/10) and the thalamus (Table 1).

**Figure 1:**
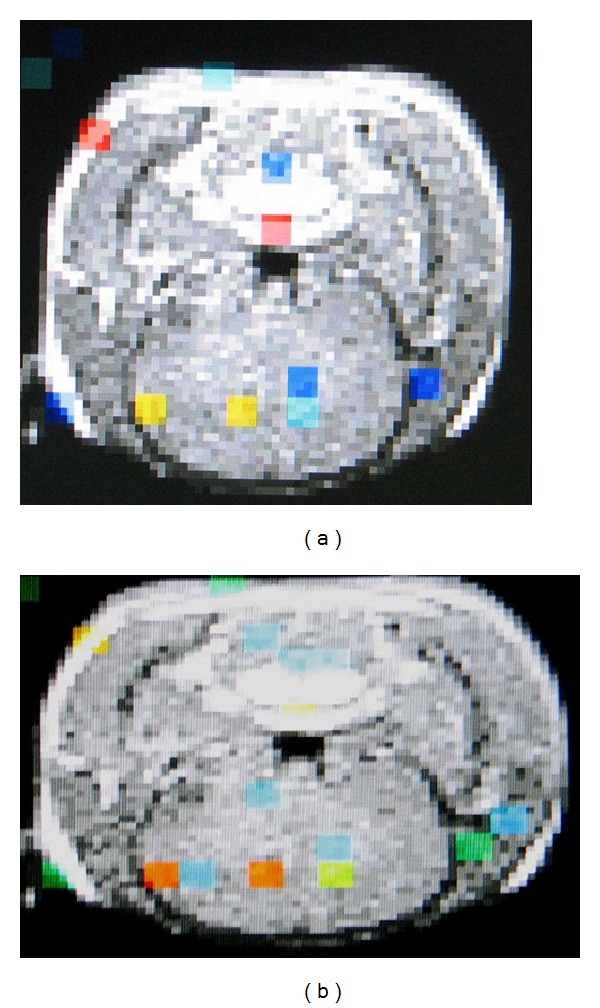
(a) Activation of the ACC, PFC, and THAL in the brain of an IBS model rat. (b) Increased activation of the ACC, PFC, and THAL with 10 mL air inflation in the balloon in the same rat.

**Figure 2:**
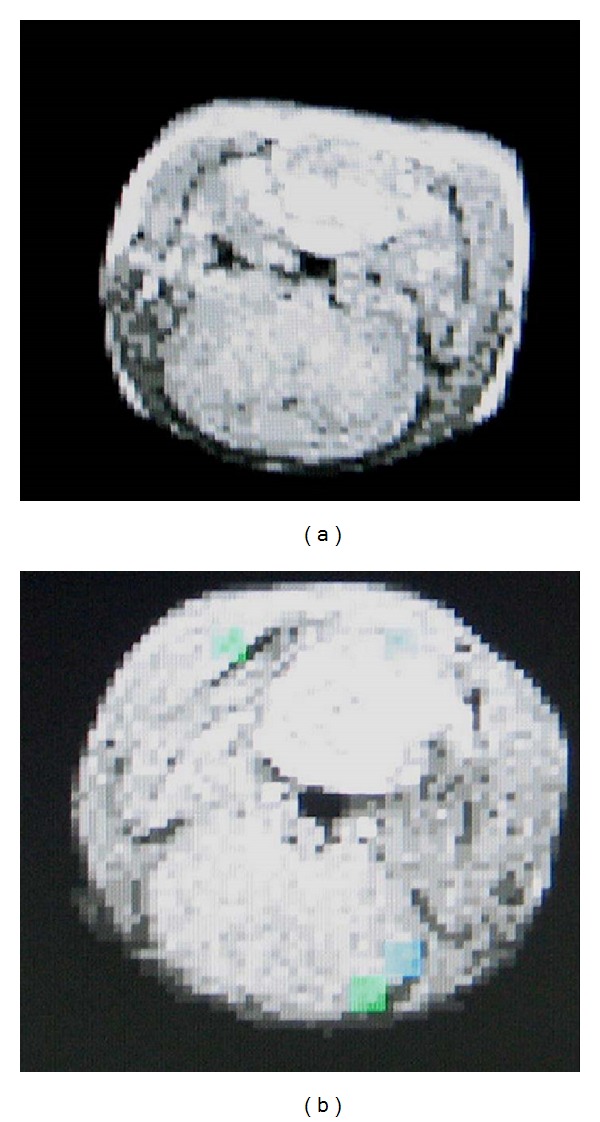
(a) fMRI of the center of visceral sensation in a rat in the control group; (b) activation of ACC after 10 mL air inflation in the same rat.

Rectal stimulation with 5 mL air balloon distension activated the anterior cingulate cortex (ACC 7/10), insula cortex (IC 7/10), prefrontal cortex (PFC 6/10), and thalamus (THAL 8/10) in most rats. Balloon distension with 10 mL air significantly activated the anterior cingulate cortex (ACC 8/10), insula cortex (IC 9/10), prefrontal cortex (PFC 9/10), and thalamus (THAL 10/10) in infant rats with IBS (Figure 1). There was no corresponding excitation in the control rats after 5 mL distension of the air balloon. Only some of the control rats had activation in the ACC (1/6) and THAL (2/6) after the balloon was inflated with 10 mL air (Figure 2).

## 4. Discussion

The basic principle of fMRI is that focal cerebral blood flow (CBF) and oxygen consumption increase when specific areas of the brain are stimulated with focal activities [13]. The technique of blood-oxygen-level dependence fMRI (BOLD-fMRI) uses endogenous hemoglobin as the contrast to obtain images by highlighting oxygen saturation changes. Such changes objectively reflect the main physiological changes of brain activities. The MR signals collected by the BOLD technique reflect oxygen saturation changes in the local capillaries and the venous beds [13].

The increase of brain tissue activities leads to a disproportionate increase of local CBF and oxygen consumption, 30% to 50% for the former, and only 5% for the latter. The net result is prolonged blood oxygen saturation in the local capillaries and venous beds. The increase in blood oxygen saturation induces prolonged T1 and T2 relaxation time in the MR images, which is similar to the findings in other studies that used PET (positron emission tomography); however, compared with PET, fMRI has the advantages of higher resolution, better precision, and noninvasiveness. In addition, fMRI does not require contrast infusion. Participants generally tolerate fMRI better, and fMRI better reflects functional changes in the central nervous system.

Currently, visceral hypersensitivity and the interaction between the brain and intestines are the major features of the suspected mechanism of IBS. The enteric nerves link the gastrointestinal system with the central and autonomic nervous systems. Colorectal balloon distension- (CBD-) induced visceral pain can be transmitted to the central nervous system through the brain-gut axis and can be detected by brain electrical activity. With the development of imaging techniques, fMRI is used in the clinical setting to reflect the brain activity directly and objectively as a means to investigate more objectively the hypersensitivity of IBS visceral pain.

ACC, IC, PFC, and THAL are parts of the cerebral center of visceral sensations. Recent research has demonstrated that the thalamus acts as the relay center in the central nervous system and receives the afferent fibers from the thalamic tract in the spinal cord to project to a higher center, such as the ACC, IC, or PFC. Researchers commonly agree that the ACC mainly processes signals of sensation related to emotions. The IC is mainly responsible for visceral sensation, including taste, smell, rectal stimulation, and other gastrointestinal sensation. PFC is the higher center of pain sensation. Some studies have used PET and fMRI to study functional diseases of the gastrointestinal tract and visceral sensation hypersensitivity.

There is a growing body of literature addressing pain sensation [14–19]. Silverman et al. [20], using PET, observed that the excitation area for rectal pain sensation in the normal population is mainly the ACC, and for IBS patients, mainly the PFC. Mertz et al. [14] conducted fMRI in 18 volunteer patients and 16 healthy volunteers with painful and painless rectal distension. They found that patients with IBS had a broader excitation area in the ACC than did the control group while receiving painful rectal distension. Contrary to previous results, their results demonstrated that the sensitivity of the brain-gut axis was increased in patients with IBS but that the pattern of activation was normal.

The animals in this study showed that rectal distension in infant rats in the control group leads to the activation of the ACC and THAL in a few rats while the balloon was being inflated. In the IBS group, the rats had activation in the ACC, PFC, and THAL. The increase of the excitation area was associated with the volume to which the balloon was inflated. This result in our experimental animal model is consistent with the observation of increased pain sensitivity in the brain-gut axis in (human) patients with IBS [14].

There are several limitations of this study including the small sample size. In addition, essential mustard oil is an irritant and could cause mucosal effects not present in IBS. However, the irritant was administered in the neonatal period. By the 30th day, the intestinal mucosal effects are repaired, but the IBS-like changes remain [12]. Previous studies have shown that intrarectal pressure associated with rectal compliance increased linearly with balloon inflation. However, we could not measure intrarectal pressure. Therefore, it is not clear whether the central changes observed are related to sensitization of pain pathways or just a higher tension in the rectal wall produced by the fixed distention volumes [21]. It is possible that the anesthesia used could have had effects on cerebral measurements. However, the fMRI could not have been conducted if no anesthesia were provided for the infant rats. This situation is similar to that in human children, so although this is a limitation of the experiments, the results likely reflect those that would be obtained in real human pediatric subjects with respect to anesthesia.

In conclusion, we have demonstrated that fMRI can be used to evaluate somatic and visceral sensation, including pain-related responses in the central nervous system. fMRI can reflect increased activation in the ACC, PFC, and THAL during rectal distension, especially in the PFC and THAL.

## Figures and Tables

**Table 1:** Activation of visceral pain-related areas in rats.

	ACC	IC	PFC	THAL
IBS model (5 mL air inflation)	1	0	1∗	1∗
IBS model (10 mL air inflation)	1	1	2∗	3∗
Control (5 mL air inflation)	0	0	0	0
Control (10 mL air inflation)	1	0	0	1

*(*P* < 0.05).

ACC: anterior cingulate cortex; IC: insula cortex; PFC: prefrontal cortex; and THAL: thalamus.
